# Degree of Handedness, but not Direction, is a Systematic Predictor of Cognitive Performance

**DOI:** 10.3389/fpsyg.2013.00009

**Published:** 2013-01-31

**Authors:** Eric Prichard, Ruth E. Propper, Stephen D. Christman

**Affiliations:** ^1^Department of Psychology, University of ToledoToledo, OH, USA; ^2^Department of Psychology, Montclair State UniversityMontclair, NJ, USA

**Keywords:** handedness, interhemispheric interaction, episodic memory, belief updating, cognitive flexibility

## Abstract

A growing body of evidence is reviewed showing that degree of handedness (consistent versus inconsistent) is a more powerful and appropriate way to classify handedness than the traditional one based on direction (right versus left). Experimental studies from the domains of episodic memory retrieval, belief updating/cognitive flexibility, risk perception, and more are described. These results suggest that inconsistent handedness is associated with increased interhemispheric interaction and increased access to processes localized to the right cerebral hemisphere.

## Introduction

Psychological research examining “individual differences” grounded in biology (in contrast with, for example, personality/temperament, or in experience) typically focuses on sex and age. Another biologically based dimension of individual differences, handedness, has received much less attention. This neglect has arisen in part because handedness research has largely been the province of neuropsychologists, and such research makes little contact with the methods and theories of mainstream psychology. This lack of contact is the product of both the idiosyncratic methods employed in handedness research (e.g., lateralized presentation of input), and the fact that, historically, research attempting to identify key functional and structural differences between left- and right-handers has produced equivocal results (other than the fact that brain asymmetry appears to be weaker and more variable in left-handers).

The purpose of this article is to acquaint the reader with a growing body of evidence identifying handedness as a robust predictor of individual differences across a number of domains. The research to be reviewed breaks with past handedness research in a critical way: instead of comparing left- versus right-handedness, it focuses on comparisons between consistent/strong-handers (CH) and inconsistent/mixed-handers (ICH). Here, we define CH as using the dominant hand for virtually all common manual activities, and ICH as using the non-dominant hand for at least one common manual activity. That is, historically, research examining individual differences in handedness focused on the effects of *direction* of hand preference on behavior, thereby comparing left versus right-handers. However, evidence has accumulated that the critical dimension on which the handedness groups differ is in *degree* (consistent versus inconsistent) of hand preference. That is, how consistently, or strongly, an individual prefers to use one versus the other hand over a wide variety of tasks may be the more appropriate indicator of cerebral organization and of behavior. In fact, we would argue here that a major reason why previous research has failed to clearly determine individual differences in handedness effects on behavior is because the measure used to define handedness has heretofore been incorrect. Instead of *direction* of hand preference being the variable of interest, it should be *degree*.

The distinction between consistent and inconsistent handedness is based on a simple median split on scores on the Edinburgh Handedness Inventory (Oldfield, 1971). Scores range from −100 (pure left handed) to +100 (pure right-handed). The population median, based on a large sample of 1595 subjects, is 80. Thus, inconsistent handedness is defined as handedness scores below 80, which is equivalent to performing at least one of the ten activities with the non-dominant hand. A summary of the population proportions of handedness is presented in Table 1.

**Table 1:** **Percentages of female and male participants, classified according to both direction and degree of handedness**.

Direction of handedness	Degree of handedness		
	Strong	Mixed	
**FEMALES**			
Right	59.3	31.0	90.3
Left	3.2	6.5	9.7
	62.5	37.5	
**MALES**			
Right	47.8	41.6	89.4
Left	2.4	8.2	10.6
	50.2	49.8	

There are two things to note about Table 1. First, right-handers tend to be consistent handed while left-handers are largely inconsistent handed. Second, consistent handedness is more prevalent among females than among males.

While it is beyond the scope of this chapter to fully explicate the mechanisms underlying the distinction between CH and ICH, key principles involve interhemispheric communication and functional access to right hemisphere processing. More consistent hand preference is associated with smaller corpus callosum size (e.g., Luders et al., 2010) and with decreased right hemisphere activation (e.g., Propper et al., 2012). Accordingly, consistent- versus inconsistent handedness is associated with decreased versus increased interhemispheric interaction and with decreased versus increased right hemisphere access, respectively. The following review will focus primarily on two task domains for which interhemispheric interaction and right hemisphere access have been implicated: episodic memory retrieval (associated with right frontal areas) and belief updating/cognitive flexibility (associated with right frontal-parietal areas), with the evidence showing that ICH exhibit superior episodic memory and increased belief updating/cognitive flexibility. Other related findings will also be presented. A summary of the findings reviewed is provided in Table 2.

**Table 2:** **Summary of research on handedness differences in memory**.

Task	Findings	Citation
Free recall of words	ICH advantage	Propper et al. (2005)
Free recall of words	ICH advantage	Lyle et al. (2008a)
Free recall of words	ICH advantage	Christman and Butler (2011)
Free recall of events from own life	ICH advantage	Propper et al. (2005)
Recall of early childhood memories	ICH advantage	Christman et al. (2006b)
Paired-associate recall	ICH advantage	Lyle et al. (2008b)
Source memory (DRM paradigm)	ICH advantage	Christman et al. (2004)
Source memory (sensory modality)	ICH advantage	Lyle et al. (2008b)
Self-reported everyday memory	ICH advantage	Christman and Propper (2008)
Self-reported dream recall	ICH advantage	Christman (2007)
Incidental memory for deeply processed words	ICH advantage	Christman and Butler (2011)
Incidental memory for shallowly processed words	No difference	Christman and Butler (2011)
Know versus remember judgments	ICH: rem > know CH: rem = know	Propper and Christman (2004)
Word recognition	No difference	Propper and Christman (2004)
Word recognition	No difference	Lyle et al. (2008a)
Face memory	ICH advantage	Lyle and Orsborn (2011)
Implicit memory	No difference	Propper et al. (2005)
Semantic memory	No difference	Propper et al. (2005)
Memory for paragraphs	ICH advantage	Prichard and Christman (2012)
Openness to persuasion	ICH more open	Christman et al. (2008)
Gullibility	ICH more gullible	Christman et al. (2008)
Belief in evolution	ICH more likely	Niebauer et al. (2004)
Magical ideation	ICH have higher levels	Barnett and Corballis (2002)
Cognitive dissonance	ICH have higher levels	Jasper et al. (2009)
Placebo effect	Larger in ICH	Christman et al. (2006a)
Anchoring effect	Larger in ICH	Jasper and Christman (2005)
Counterfactuals	ICH produce more	Jasper et al. (2008)
Ambiguous figures	ICH higher reversal rate	Christman et al. (2009)
Ambiguous words	Greater activation in ICH	Sontam and Christman (2012)
Musical preferences	Greater preference for obscure genres in ICH	Christman (2013)
Sensation seeking	Higher levels in ICH	Christman (2011a)
Consumer loyalty	Lower levels in ICH	Lanning and Christman (2010)
Right Wing Authoritarianism	Lower levels in ICH	Christman (2008)
Sense of disgust	Stronger in CH	Christman (2012)
Risk perception	ICH more loss averse	Christman et al. (2007b)
Sunk cost effect	Higher levels in ICH	Westfall et al. (2012)
Taking others’ perspectives	ICH are better	Sontam et al. (2005); Lanning and Christman (2010)
Sleep architecture	ICH: shorter latency, increased time in NREM	Propper et al. (2007)

*ICH, inconsistent handed; CH, consistent handed*.

We are not arguing that the reader should become a “handedness” researcher. Instead, we are encouraging researchers to include degree of handedness as a variable in their designs, much like many already do with sex and/or age. At the very least, including handedness as a variable in analyses would move variability out of the omnibus error term and into a specific effect term, thereby providing increased statistical power to detect other effects of primary interest. At best, systematic individual differences as a function of handedness and concomitant gradations in interhemispheric interaction and in right hemisphere access, could prove to be a useful construct in the development and testing of domain-specific theories.

## Handedness and Memory

Some of the most robust findings demonstrating the effects of handedness as an individual difference variable come from the domain of memory research. This work initially relied on predictions made by the Hemispheric Encoding and Retrieval and Asymmetry (HERA) model (Tulving et al., 1994). They reported that, for semantic memory tasks, brain activity at both encoding and retrieval were lateralized to the left hemisphere. In contrast, for episodic memory, activation at encoding versus retrieval was lateralized to the left versus right hemispheres, respectively. This finding raised the possibility that (i) episodic memory relies on interhemispheric interaction (necessary to integrate left hemisphere encoding with right hemisphere retrieval) to a greater extent than does semantic memory (left hemisphere encoding and retrieval); (ii) individual differences in interhemispheric interaction would be reflected in individual differences in memory ability, and (iii) individual differences in degree of hand preference, being associated with individual differences in interhemispheric interaction, would therefore also be associated with individual differences in memory performance. Specifically, inconsistently handed individuals, having increased interhemispheric interaction, possibly mediated via greater corpus callosal connectivity, would demonstrate superior episodic, but not semantic, memory.

Supporting the hypothesis, Propper et al. (2005) found that ICH outperformed CH on an episodic memory task involving word list recall. Interestingly, there was no significant difference between the handedness groups on a word fragment completion task used as a test of semantic memory. Handedness differences are not typically found in recognition memory; however, in another test of episodic memory (e.g., Christman and Propper, 2001), Propper and Christman (2004) found that, despite equal levels of recognition memory, ICH are more likely to report explicitly episodically “remembering” an item while CH are more likely to report merely semantically “knowing” that they saw the item before.

The findings that ICH have superior episodic recall abilities and show a greater tendency than CH to make “remember” judgments raises the possibility that handedness differences in episodic memory may reflect underlying differences in source memory. Three findings examining handedness differences in false alarms, using both laboratory based and real-world memories, support this notion. First, Christman et al. (2004) demonstrated that ICH are less likely to report having a false memory for the critical lure in by the Roediger and McDermott (1995) paradigm, suggesting that the source of memories may be more available in the ICH relative the CH. Second, Lyle et al. (2008b) tested source memory for words that participants had originally either read or heard; again, relative to CH, ICH were better at remembering whether the original presentation of items has been visual or auditory. Finally, Lyle et al. (2008a) also reported fewer false alarms in ICH.

These “snapshot” handedness effects on memory-that is, of superior episodic memory among ICH relative to CH− have been extended in investigations of handedness and memory effects across the lifespan. Christman et al. (2006b) reported that ICH experience an earlier offset of childhood amnesia, and therefore a younger age for their earliest childhood memory. Lyle et al. (2008b) obtained an ICH advantage on a recall task in a sample of middle aged adults, but not with a sample of older adults. Lyle et al. (2008b) proposed that as people age, the corpus callosum degenerates, thus attenuating the ICH advantage. Specifically, the decline in memory from middle- to older-aged adults was larger in the ICH, consistent with a callosal contribution to episodic memory. Finally, Kempe et al. (2009) found that ICH were more easily able to acquire foreign vocabulary words in adulthood. Although vocabulary recall in adulthood may involve both episodic and semantic memory processes, this finding suggests that individual differences in handedness may account for some between individual variability in language acquisition.

Findings of superior episodic memory in ICH relative the CH extend beyond artificial, laboratory information. For example, Propper et al. (2005) demonstrated an ICH advantage for autobiographical, self-reported events that occurred outside the laboratory, and Christman et al. (2006b) reported an ICH advantage for earliest childhood memories. Christman and Propper (2008) found that ICH reported fewer memory problems in everyday life, especially in the domains of task monitoring and conversation. Christman (2007) reported that ICH remember more dreams and report more frequent déjà vu experiences. Prichard and Christman (2012) found that the ICH advantage in memory extends to recall of paragraph-level material, although the ICH advantage was larger for males than for females. Finally, Lyle and Orsborn (2011) reported superior face memory in ICH.

It is important to point out that in the studies reviewed above, most compared ICH with consistent *right*-handers. Given that consistent-left-handers are only about 1–3% of the population (Lansky et al., 1988), studies comparing ICH with consistently right and consistently left handed individuals are time consuming, difficult to conduct, and therefore infrequent. However, Lyle et al. (2012) collected a large sample of consistent-left-handers in order to determine whether it is consistent handedness *per se* that is associated with less or episodic memory, or if this effect is specific to consistent-*right*-handedness. Importantly, ICH outperformed CH, on an episodic memory task, regardless of the *direction* of CH hand preference; that is, regardless of whether CH were left- or right-handed, ICH performed better.

## Handedness and Belief Updating/Cognitive Flexibility

Ramachandran (1995) hypothesized that the left hemisphere is important for maintaining our current beliefs about the world, while the right hemisphere acts as an anomaly detector and is sensitive to information inconsistent with those beliefs. This suggests a possible role for interhemispheric connectivity in the belief updating process. When something challenges pre-existing beliefs, it may be the right hemisphere’s job to notice the inconsistency and communicate it to the left hemisphere. Since belief updating may be considered, more broadly, an example of cognitive flexibility, further studies have also looked at numerous DVs which, taken together, may be considered measures of cognitive flexibility. It is to the literature investigating a possible relationship between handedness and belief updating/cognitive flexibility which we now turn.

Niebauer et al. (2004) found that consistent-handers are more likely to report holding young-earth creationist beliefs. The authors argued that, because children typically hold creationist views at some point (Evans, 2000), the retention of such beliefs is the result of a failure to update beliefs about human origins in light of new evidence. Similarly, Christman et al. (2008) reported that ICH are more open to persuasion. At the same time, however, they found that ICH were also more gullible, showing greater susceptibility to the “Barnum effect.” This finding may be related to that of Barnett and Corballis (2002), who reported that ICH were more prone to magical ideation (i.e., beliefs in ESP, UFOs, astrology, etc). Thus, CH are more resistant to belief updating, and are therefore less likely to alter their views based on little evidence.

Once researchers obtained the basic finding that degree of handedness predicts the tendency to update one’s beliefs or, to frame it differently, degree of handedness predicts resistance to information challenging pre-existing beliefs, the handedness paradigm has been applied to several areas for which the belief updating/cognitive flexibility process is relevant. For example, it has been applied to cognitive dissonance (Jasper et al., 2009), who conducted a study in which participants were given false personality profiles. In the experimental condition, participants were told their profiles indicated high levels of sexism. When asked to judge a fictional sex based discrimination suit, ICH who had been told they were sexist awarded higher payouts than CH, indicating greater cognitive dissonance in ICH. Handedness differences have also been obtained in the magnitude of placebo effects, with ICH exhibiting much larger placebo effects than CH (Christman et al., 2006a). Thus, handedness may be a variable of interest for researchers examining how belief affects treatment outcomes, or for researchers who want to reduce the error term in clinical trials that necessarily include a placebo condition.

As stated at the beginning of the section, belief updating could arguably fall under the broader area of cognitive flexibility. The empirical evidence indicates handedness does indeed predict cognitive flexibility as measured by a surprising variety of DVs. Starting with research on basic heuristics, Jasper and Christman (2005) found that inconsistent-handers were less susceptible to anchoring on a task that asked participants 12 factual knowledge questions after exposing them to unhelpful high or low anchors. Resisting anchors in such a situation may require one to hold multiple representations, a process requiring considerable cognitive flexibility. In the area of counterfactual reasoning, Jasper et al. (2008) found that, when asked to come up with counterfactual alternatives to various scenarios, ICH produce more upward counterfactuals and downward counterfactuals. Research on more basic perceptual processes shows that ICH can more easily update their perception of ambiguous figures (Christman et al., 2009) and that ICH more readily fall for a sensory illusion in which a participant comes to “feel” taps on a fake arm (Niebauer et al., 2002). During investigations of semantic flexibility, ICH showed a greater tendency to switch between subcategories when asked to name as many animals as they could (Sontam et al., 2009) and had an easier time accessing “weak” associates of ambiguous stimulus words than consistent-handers did (Sontam and Christman, 2012). ICH have also been shown to be more creative, measured via divergent thinking, compared to CH (Shobe et al., 2009).

As with the handedness and memory paradigms, there has been an interest in whether these cognitive flexibility effects generalize beyond the realm of interesting experiments. What does it mean to say inconsistent-handers are more cognitively flexible outside of an experimental context? As it turns out, handedness predicts certain kinds of esthetic judgments, with ICH showing more appreciation for self-referential works by M.C. Escher (Niebauer and Garvey, 2004) and for a wider variety of musical genres (Christman, 2013) than CH. Further, consistent-handers are less sensation seeking (Christman, 2011a), exhibit greater consumer brand loyalty (Christman and Lanning, 2012), have greater disgust sensitivity (Christman, 2012), and score higher on measures of Right Wing Authoritarianism (Christman, 2008) than ICH.

Perhaps of the greatest practical relevance, the link between handedness and cognitive flexibility is of potential clinical relevance. Consistent-handers are more likely than inconsistent-handers to ruminate (Niebauer, 2004), to display eating disorder symptomatology (Christman et al., 2007a), and to show higher levels of body dysmorphia (Christman, 2011b).

## Miscellaneous Handedness Findings

While the memory and cognitive flexibility literatures are the most well developed of the literatures investigating degree of handedness as an individual difference variable, it is worth mentioning several empirical studies that have branched out beyond these two major areas. Although much remains to be explained about what underlies the following findings, it is hoped that there will be something of interest to researchers from across the discipline of psychology.

Several studies looking at emotion and risk perception have uncovered evidence of handedness effects. Propper et al. (2010) reported that ICH demonstrated increased negative affect across a wide variety of emotional states, compared to CH, although only feelings of “anger” were significantly greater in ICH. Christman et al. (2007b) found that, when making risky decisions, inconsistent-handers reported being more influenced by the perceived risks of a behavior and consistent-handers reported being more influenced by the perceived benefits. Westfall et al. (2012) found that inconsistent-handers showed more inaction inertia and a greater sunk cost effect unless it was made clear that staying on a particular course would definitely result in a greater loss than abandoning it. Once it was clear that inaction would definitely result in a greater loss, there was a reversal with inconsistent-handers showing less inaction inertia. Finally, Bhattacharya et al. (2012) found that selectively activating the right hemisphere via Schiffer goggles increased the tendency for inconsistent-handers to focus on risks and consistent-handers to focus on benefits. It may be that these findings are related to a potential right hemisphere role in negative affect/withdrawal motivational states.

Handedness has also been used as a variable in traditional self-other/person perception paradigms. ICH seem to have an easier time taking other perspectives into account (Sontam et al., 2005; Lanning and Christman, 2010) and have better memory for counter-stereotypical information (Christman and Sterling, 2009). Additionally, sex and race effects on the Implicit Association Test (IAT) are modulated by handedness (Christman and Sahu, 2012). For example, the weakest stereotyping was exhibited by ICH European Americans and by CH African-American males.

We will wrap up the present review with several additional findings that are not yet part of any broad research program, but which may prove to be promising leads in the future. While investigating possible associations between handedness and sleep architecture, Propper et al. (2004) found that ICH had shorter sleep latency and spent more time in NREM, although Propper et al. (2007) also obtained evidence that *consistent*-left-handers spend more time in NREM and less time in REM than *consistent*-right-handers, thus raising the possibility that *both* strength and direction of handedness should be considered when researching sleep. Christman (1993) discovered a compelling example of how degree of handedness may be related to preferences for certain motor tasks when he surveyed musicians and found that ICH were more likely to play instruments that require temporally integrated bimanual motor actions.

In conclusion, the studies reviewed above demonstrate a robust and systematic effect of degree of handedness in two well defined domains; episodic memory retrieval and belief updating/cognitive flexibility, and in other areas as well, including emotion and sleep architecture. It is hoped that this review will inspire a wider body of psychology investigators to incorporate this long neglected and misunderstood dimension of human individual difference into their research.

## Conflict of Interest Statement

The authors declare that the research was conducted in the absence of any commercial or financial relationships that could be construed as a potential conflict of interest.
